# Abdominal Pain in Acute Rheumatism

**Published:** 1904-06-18

**Authors:** 


### ABDOMINAL PAIN IN ACUTE RHEUMATISM.

The manifestations of rheumatism in early life are both less pronounced and more varied than would seem to be the case in the adult. It is rare, for example, in a child to see the acute polyarthritis which distinguishes an ordinary atrack of rheumatic fever. On the other hand, a great variety of events occurring in childhood are now brought within the rheumatic series, and the recognition of the true nature of these is a matter of the first importance. It is only when this recognition is effected that suitable treatment is secured. And, what is perhaps of even more importance, the same circumstances are necessary to secure for the child the early and complete rest which is the best prophylactic influence against the grave complications of carditis and endocarditis. It is the thorough study of rheumatism in childhood which affords the most hopeful prospect of diminishing the number of victims of cardiac valve disease. Pain in the epigastrium, or more widely spread over the abdomen, has been referred to by several writers as a manifestation of rheumatism in childhood, and more recently Dr. S. Vere Pearson[1] has studied the subject with peculiar care. He finds that the pain is nearly always situated in the upper half of the abdomen, and is rarely referred solely to the umbilicus. It usually commences close to the hypochondriac margin of the ribs in the mid-clavicular line, and passes either towards the umbilicus or straight across the epigastrium ; occasionally it is purely epigastric and is in the middle line. The pain is paroxysmal, and after lasting several minutes disappears, only, however, soon to recur. It is apt to be brought on by exercise or excitement. The pain is not associated with evidences of gastro-intestinal disturbance, and there is neither superficial nor deep abdominal tenderness. Dr. Pearson relates a number of interesting cases in support of his description, and it is noteworthy that in several of these, whilst the abdominal pain was the sole ground of complaint on the patient's part, careful examination detected other and conclusive signs of rheumatism, such, for example, as subcutaneous nodules. Dr. Pearson notes aspirin as a useful remedy in the condition.

[1] Brit. Med. Jour., May 14, 1904.

